# Supplementary material for: The Middle Part of the Plucked Hair Follicle Outer Root Sheath Is Identified as an Area Rich in Lineage-Specific Stem Cell Markers
Source: Biomolecules. 2021 Jan 25;11(2):154. doi: 10.3390/biom11020154 (PMC7912066; doi:10.3390/biom11020154)
Supplement: Supplementary file 1 [file biomolecules-11-00154-s001.pdf]

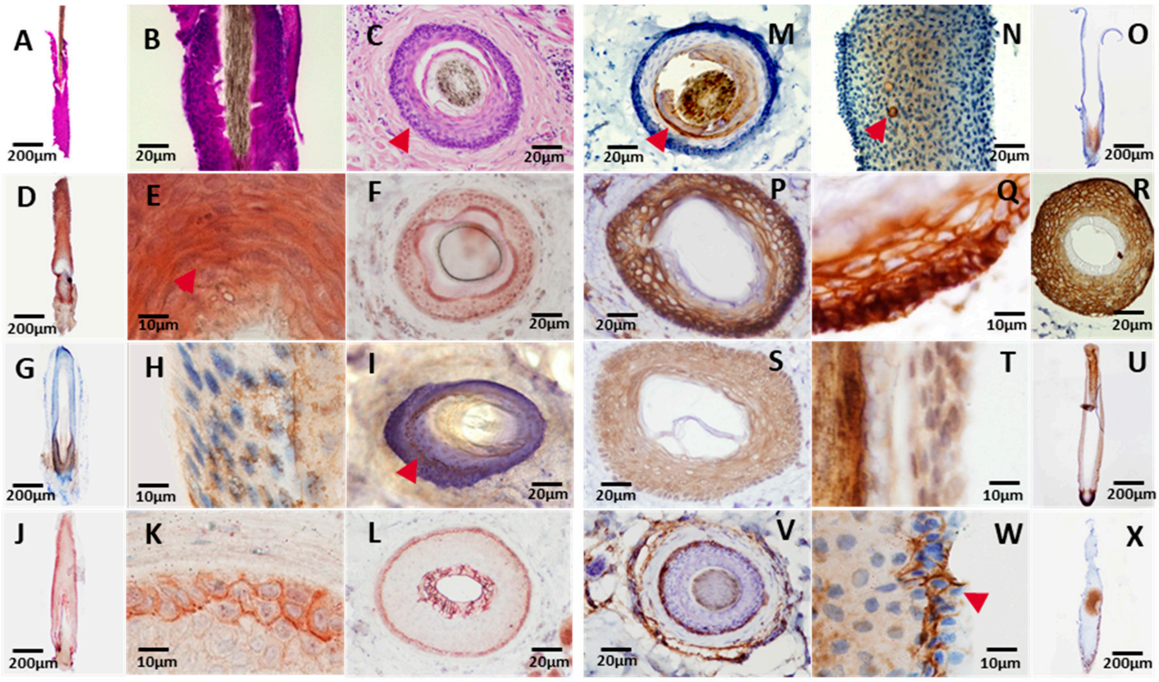

Figure S1. Histological and Immunohistochemistry of the biomarkers in human plucked and *in situ* hair follicles. Paraffin-embedded human plucked hairs or scalp tissue were immunohistochemically stained with various antibodies capturing melanocyte and stem cell biomarkers. Vertical and horizontal sections of a human terminal anagen hair follicle from both plucked (outer line) and *in situ* (center line) positions. The follicles were stained with Hematoxylin & Eosin (H&E), anti- sAC, anti-Nestin, anti- CD44, anti- CD133, anti- CD34 antibodies. The immunochemical reaction was visualized by using either DAB (3,3'-diaminobenzidine, brown) or AEC (3-Amino-9-Ethylcarbazole, Red), and the nuclei were counterstained with methylene blue (for DAB coloration), and hematoxylin (for AEC coloration). (**A,B,C**). H&E staining clearly showed the whole anatomic structure of the hair follicle, with the dark brown hair shafts, cuticularized IRS (inner root sheath), compact ORS and dermal papilla in the plucked hair follicle, as well as the perifollicular dermal sheath (pointing arrow) and dermal matrix in the scalp. (**D,E,F**) The sAC expression was displayed as an intensive signal dispersed in the ORS cells, distinguished from the dermis with point-like expression in the fibroblasts. (**G,H,I**) CD34 was expressed in the outer layer of the basal ORS region with a clear demarcation towards the dermal sheath, and a distinctive kidney-shaped population of CD34-positive cells in the mid portion of the plucked hair follicle RS. (**J,K,L**) Nestin was expressed between the ORS and IRS, within the lower portion of the plucked hair follicles (pointing arrow). (**M,N,O**) Hair follicle ORS gave off a remarkably intensive CD44 signal, with a clear distinction towards the dermal matrix. (**P,Q,R**) CD133 showed dispersive expression in the ORS cells and it was not expressed in dermis.

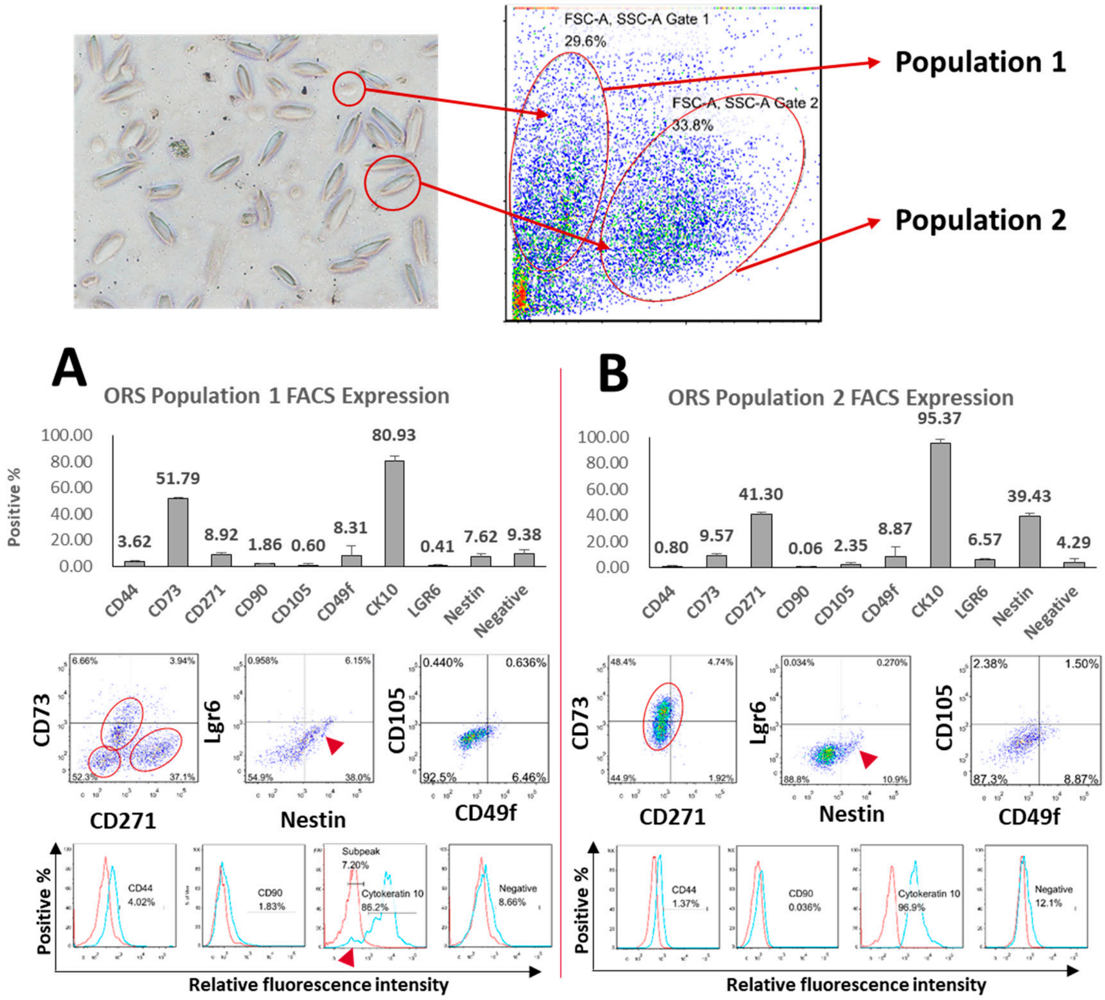

Figure S2. Expression Profile of single cell suspension of hair follicle ORS of two cell populations. The FACS data were re-analyzed after separating the cells into two subpopulations on the basis of two separate FSC/SSC gates: Gate 1 (subpopulation 1, relative size, scatter from-to, for the small round cells) and Gate 2 (subpopulation 2, relative size, scatter from-to, for the large spindle-shape cells). (**A**) Cells from Population 1 expressed CD44 (3.62±0.72%), CD73 (6.37±1.64%), CD271 (38.1±5.03%), CD90 (1.86±0.70%), CD105 (1.24±0.80%), CD49f (Integrin alpha 6, 7.76±5.91%), Cytokeratin 10 (80.93±3.76%), Lgr6 (0.93±0.36%), Nestin (33.8±5.79%), MSC Negative Markers (9.37±4.73%). (**B**) Cells in Population 2 expressed CD44 (0.801±0.40%), CD73 (45.4±2.16%), CD271 (2.53±0.81%), CD90 (0.05±0.04%), CD105 (0.17±0.18%), CD49f (Integrin alpha 6, 7.88±7.09%), Cytokeratin 10 (95.36±1.89%), Lgr6 (0.06±0.02%), nestin (7.27±2.61%), MSC Negative Markers (4.29±5.52%).

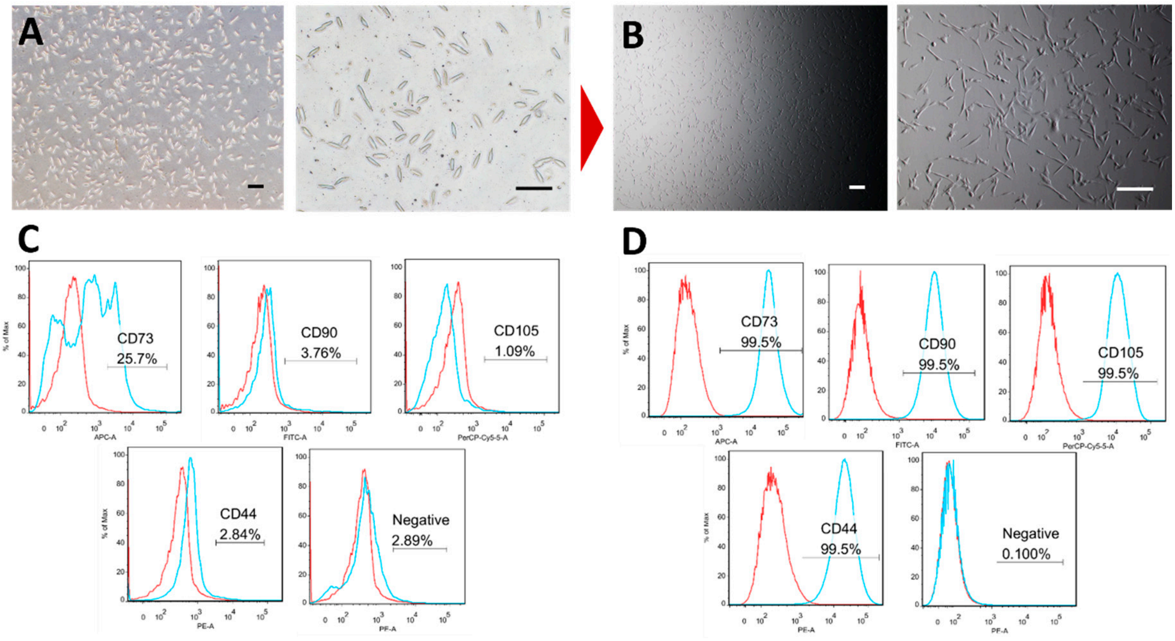

Figure S3. Comparison of ORS single-cell suspension and MSCORS isolated and cultivated based on explant outgrowth method. Primary cell culturing using ORS single cell suspension and MSCORS migration method were compared in cell morphology and attachment. (**A**) ORS single cell suspension was obtained using the digestion method described in the study. Briefly, non-invasively removed hair follicles were plucked, intensively washed, dermal papilla excised, and completely digested into single cells using 0.04% Trypsin/0.03% EDTA. This cell suspension was seeded onto a cell culture flask for primary cultivation. After 7 days in culture, cells showed no sign of cell attachment or proliferation. (**B**) MSCORS cells were obtained using the migration method presented previously [14]: hair follicles were non-invasively plucked, intensively washed, the dermal papilla was excised, and the prepared follicles were seeded onto a Transwell™ porous membrane (Corning Inc., Lowell, USA), which allows the cells from the ORS follicle to migrate onto the nylon mesh, attach and form a cell monolayer on the porous membrane. Upon reaching confluence, the cell monolayer was split into single cells using 0.04% Trypsin/0.03% EDTA, and seeded onto a cell culture flask for primary cultivation. After 7 days, MSCORS cells attached to the plastic surface, displayed dendritic bi- or tri-polar morphology with rapid proliferation. (**C**,**D**) Representative FACS histograms of MSC biomarker expressions in ORS single cell suspension and in MSCORS method. In ORS cell suspension, following markers were used: CD44 (2.84%), CD73 (25.7%), CD90 (3.76%), CD105 (1.09), and MSC Negative Markers (2.89%). On the other end, MSCORS cultures returned following marker profiles: double staining in CD44 (99.3%) and negative markers (CD34, CD11b, CD19 PE, CD45 PE, HLA-DR, <0.001%) with CD73 (99.8%), CD90 (99.4%), CD105 (99.7%) .

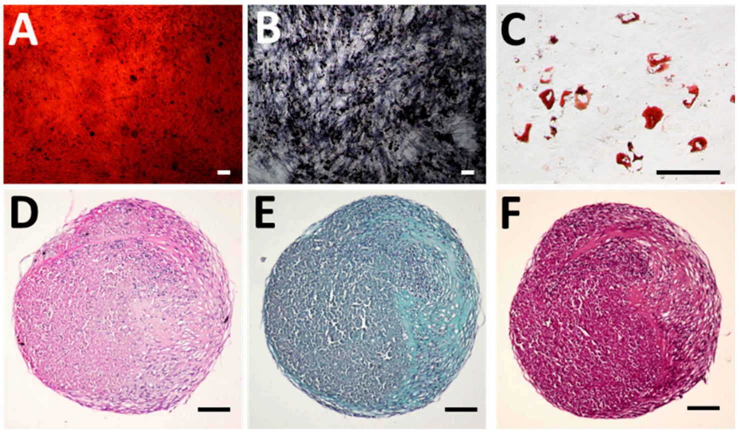

Figure S4. Differentiation potentials of mesenchymal stem cells isolated from the plucked hair follicle outer root sheath (MSCORS). MSCORS cells were obtained using the aforementioned migration method, and differentiated towards SC tri-lineage: osteogenic (**A**,**B**), adipogenic (**C**) and chondrogenic (**D-F**). Briefly, for osteogenic differentiation, MSCORS were plated at cell density of 2x10^4 cells/cm^2 and stimulated for 21 days in osteogenic medium containing 200nM Dexamethasone, 50ug/ml Ascorbic Acid, 10mM β-glycerophosphate, 10% Fetal Bovine Serum, 2mM L-Glutamine in DMEM (Low Glucose, ThermoFisher Scientific, Darmstadt, ThermoFisher Scientific, Darmstadt, Germany). Calcium deposition (**A**) was stained using 2% Alizarin Red (Carl Roth GmbH, Karlsruhe, Germany) and alkaline phosphatase (ALP) activity (**B**) was detected using BCIP/NBT (5-Bromo-4-chloro-3-indolyl phosphate/Nitro blue tetrazolium) substrate (Sigma-Aldrich Chemie GmbH, Steinheim, Germany). For adipogenic differentiation, MSCORS were seeded at 2x10^4 cells/cm^2, and induced for 21 days in StemPro™ Adipogenesis Differentiation Kit (ThermoFisher Scientific, Darmstadt, Germany), and the lipid vesicles (**C**) were stained by 0.18% Oil Red (Carl Roth GmbH, Karlsruhe, Germany). For chondrogenic differentiation, 2.5x10^5 MSCORS cells were centrifuged into a pellet and differentiated in chondrogenic medium containing 10ng/ml TGF-β1, 10ng/ml BMP-4, 1% Human Serum, 1% ITS Premix, 2mM L-Glutamine, 50ug/ml Ascorbic Acid, 50ug/ml Na Pyruvate, 1% Non-essential AA in DMEM (Low Glucose, ThermoFisher Scientific, Darmstadt, Germany). The differentiated pellet was fixed, embedded and histologically sectioned and stained in H&E (**D**), Alcian Blue (**E**) and Safranin O (**F**) (Carl Roth GmbH, Karlsruhe, Germany). Scale bar: 100µm; magnification: (**A**,**B**) 4x, (**C**) 20x, (**D-F**) 10x.
